# Proportion and associated factors of maternal complications of cesarean sections among mothers who deliver at Bahir Dar City Public Specialized Hospitals, Bahir Dar, Ethiopia

**DOI:** 10.1186/s12905-023-02388-y

**Published:** 2023-05-06

**Authors:** Hiwotemariam Alemu, Zeamanuel Anteneh Yigzaw, Lakachew Asrade, Bantayehu Nega, Amare Belachew

**Affiliations:** 1 Department of obstetrics and gynecology, school of Medicine, college of medicine and health sciences, Bahir Dar University, Bahir Dar, P.O.Box 79, Ethiopia; 2 Department of Health promotion & Behavioral science, school of public health, college of medicine and health sciences, Bahir Dar University, Bahir Dar, Ethiopia; 3 Department of Pediatrics and Child Health Nursing, College of Medicine and Health Sciences, Bahir Dar University, Bahir Dar, Ethiopia

**Keywords:** Maternal complication, Cesarean section, Bahir Dar

## Abstract

**Introduction:**

Cesarean delivery carries both short term and long-term maternal complications. Eventhough it’s being a public burden, the proportion of complications and underlying risk factors are not studied well in our setup. This study aimed to assess the proportion and associated factors of complications of cesarean sections among mothers who delivered at Bahir Dar city public specialized hospitals, Bahir Dar, Ethiopia 2021.

**Methods:**

A cross-sectional study was conducted at two specialized Hospitals in Bahir Dar city, Ethiopia. The sample size was 495 mothers who had cesarean section in the time period from January 1, 2020 to December 30, 2020. Checklist was used to retrieve information from the patient medical document. Study population was selected from the operation registration book. Systematic sampling was used after arranging the study frame based on date of operation. Both bivariable and multivariable logistic regression was done. In multivariable logistic regression variables with p value < 0.05 at 95% confidence interval were significantly associated with outcome variable.

**Result:**

Overall maternal complication rate was 44.04% (95% CI: 39.6–48.5). Living in rural setting (AOR = 4.247,95%CI: 2.765–6.522), having one or more obstetric complication (AOR = 1.913,95% CI: 1.214–3.015), cesarean section done at Second stage of labor (AOR = 4.358,95%CI: 1.841–10.317), having previous cesarean section (AOR = 3.540,95%CI: 2.121–5.910), emergency operation (AOR = 2.967,95%CI: 1.492–5.901), duration of surgery taking more than 60 min (AOR = 3.476,95%CI: 1.521–7.947) were found to be significantly associated with maternal complications.

**Conclusion:**

The magnitude of maternal complication of cesarean section was higher than most studies. Living in rural setting, having obstetric complications, previous cesarean scar, emergency surgeries, operation done in second stage of labor and prolonged duration of surgery are important predictors of maternal complication. Therefore, we recommend timely and adequate progress of labor evaluation, timely decision for cesarean delivery and vigilant care in post-operative period shall be conducted.

## Introduction

Cesarean delivery (CD) is one of the most common major surgical procedures performed in an operating room. It can be defined as the delivery of the fetus, membranes, and placenta from an intact uterus through an abdominal incision after fetal viability. Cesarean section rate is different across countries, ranging from 6% in the least developed regions to 27.2% in more developed regions [1]. In Africa, cesarean deliveries accounted for 8.8% of all births [2]. For better maternal and perinatal health, the World Health Organization (WHO) has proposed an incidence between 10% and 15% as a target [3]. The overall pooled prevalence of Caesarean sections in Ethiopia was 29.5% [4].

Cesarean delivery can be performed for maternal-fetal, fetal, and maternal indications. Non-reassuring fetal heart rate pattern (NRFHRP), mal-presentation, malposition, cephalo-pelvic disproportion (CPD), obstructed labor, multiple pregnancy, previous CS, failed induction, and antepartum hemorrhage are most common indications in Ethiopia [4].

Cesarean section is a common and lifesaving procedure. However, it carries both short-term and long-term fetal and maternal complications [1, 4]. Different studies reported that maternal morbidity rates increased with cesarean section compared with vaginal delivery [5–8]. In western studies done over a 10-year period studying intraoperative and postoperative complications of CS, the overall maternal intraoperative complication rate was 14.8%, with a postoperative morbidity rate of 35.7% [9]. In a study done in Ethiopia, the overall maternal complications of CD were found to be 38.2%. One study done in Jimma Hospital reported an overall cesarean section maternal morbidity of 20% [10] .

CD increases the risk of maternal morbidity, including hemorrhage, anesthetic complications, obstetric shock, cardiac arrest, acute renal failure, venous thromboembolism, surgical site infection, puerperal sepsis, hematoma, and maternal mortality [2, 6, 8, 11–13]. In developed countries, febrile morbidity, puerperal sepsis, postpartum hemorrhage, surgical site infection, maternal mortality, and severe anemia were the most common CS complications [14, 15].

Though little is known about factors contributing to adverse CS complications, the following are some of the documented risk factors for CS-related complications: prolonged labor, PROM, chorioamnionitis, placental abruption, preeclampsia, eclampsia, and surgeon’s experience, the competence of the center, surgical technique, and type of anesthesia. Maternal factors like age, residency, obesity, and DM are also some of the factors for post-CS complications [8, 11].

Not all but most complications can be preventable based on the modifiability of the underlying risk factors and better readiness and preparation. It will result in a better patient outcome and satisfaction by allowing health care provider to prepare on preventive mechanisms for common complications and avoid modifiable risk factors. The result will also help policy makers, program planners and maternal health service providers to propose interventions for a better maternal health and outcome. There is limited evidence on maternal complications of cesarean delivery in Ethiopia. Therefore this study aimed to assess proportion and associated factors of maternal complications of cesarean delivery.

## Methods

### Study design

A Facility based cross sectional study design was conducted.

### Study area and period

The study was conducted from September 1 to October 30, 2021 G.C on cesarean sections done from January 1, 2020 to December 30, 2020 G.C. It was held in Bahir Dar city which is the capital city of Amhara region. It is located 565 km North-West to Addis Ababa. Two specialized hospitals were selected for this study namely Tibebe Ghion specialized hospital and Felege Hiwot Comprehensive Specialized hospital. These are two of the popular governmental hospitals found in Bahir Dar. FHRH has big maternity ward which possesses around 74 beds. There are around 4500 deliveries per year. There are 5 obstetricians, 10 residents and 52 midwives currently working in the department of obstetrics. TGSH has around 70 beds in Obstetrics & Gynecology ward. There are about 3400 deliveries per year. There are 20 obstetricians, 56 residents and 72 midwives currently working in the department. Both major and minor gynecologic and obstetric procedures are performed in both hospitals; cesarean section being the most commonly performed procedure.

#### Source population

Mothers who gave birth at TGSH and FHCSH from January 1,2020 to December 30, 2020 GC.

#### Study population

Randomly selected mothers who gave birth at TGSH and FHCSH from January 1, 2020 to December 30, 2020 through cesarean section.

#### Inclusion and exclusion criteria

##### Inclusion criteria

- Women who had cesarean section in the study period and whose document retrieved from the archive.

##### Exclusion criteria

- Women who gave birth during the study period but whose full information couldn’t be archived.

#### Sample size determination and sampling technique

Sample size was calculated using Epi info for a single population proportion formula with the assumption of 95% level of confidence and 3% margin of error.

Sample size was calculated using the study done in Arba Minch having the most common complication as wound infection (P) − 12% to get the most manageable maximum sample size.

By using the formula:

$$n = \frac{{{{\left( {Z\alpha /2} \right)}^2}p\left( {1 - p} \right)}}{{{d^2}}}$$

n-Sample size.

Zα/2-1.96,

d = Margin of error (expressed in decimal) = 0.03 and.

P (percentage from previous study in decimal) = 0.12.

N = 450, Adding 10% non-response rate, total sample size = 495.

The total sample size for this study was 495.

The number of cases shared from TGSH and FHCSH were determined based on the total number of CS performed in each hospital in the selected time period. Study population were selected from the operation registration book. Systematic sampling was used after arranging the study unit according to the date of operation. The first case was selected with lottery method from the first 5 cases.

Total number of CS in FHCRH in 1 year is 1598 (69.9%), in TGSH 686 (30.1%).

Total number of sample from FHCSH and TGSH was 346 and 149 respectively.

For selection; K (Skip interval).

N (total number of operation) and.

n (sample size).

(K) = N/n.

For FHCRH 1598/346 = 4.6 / every fifth case.

TGSH 686/149 = 4.6 / every fifth case.

#### Study variables

##### Dependent variable

Maternal complications of cesarean section (yes or no).

##### Independent variables

Socio demographic factors (age and residence), Obstetric factors (Parity, Gestational age, Previous CS, Status of the labor), Obstetric complications (Pre-eclmpsia/eclampsia, APH, PROM/chorioamnionitis, Mal-presentations, Multiple gestation, Macrosomia, Prolonged labor), Operation related factors (Type of CS, Anesthesia, Duration of operation), Facility factors (ANC follow up) and Comorbidities (DM, HTN, Obesity).

#### Operational definition

##### Maternal complication of cesarean section

Includes the presence of at least one of the intra-operative surgical complication or postoperative maternal complication. Having atleast one complication coded as “1” and no complications as “0” was used as the dependent variable.

##### Urban

places having high population (at least 2000) and built-up infrastructure like water, electricity, markets, educational and administrative centers.

**Postoperative anemia**- Hemoglobin < 11 mg/dl.

Mild anemia – hemoglobin 10–10.9 mg/dl.

Moderate anemia – hemoglobin 7–9.9 mg/dl.

Severe anemia – hemoglobin < 7 mg/dl.

#### Data collection tool and procedures

Data collection was from client chart done by a designed, closed ended checklist containing the important preoperative, intraoperative and postoperative data. Data were collected by five midwives and five residents working at OBS/GYN department and they were supervised by a senior resident. For this one day training was given about the objective of the research, how to carry out data collection, how to use the checklist, how to review the patient chart and quality control.

#### Data processing and analysis

Data were coded on pre-arranged coding sheet. Data entry was done using Epi Data 3.1 and exported to SPSS 23.00 version for analysis. Descriptive statistics was computed and presented in the form of texts and tables. Bivariable analysis was used to determine the association between different factors and the outcome variables. Those variables which were significant on bivariable analysis (P-value < 0.2) were entered to multivariable logistic regression analysis. The variables with p < 0.05 in multivariable regression were considered as statistically significant associated factors. The strength of association between dependent and independent variables was determined using odds ratio (OR) with 95% confidence interval (CI). Model fitness was checked with Hosmer & Lemeshow model goodness of fit test (with p value 0.881). Multicollinearity test was done and all factors had a variance inflation factor less than 10.

#### Data quality control

Data collectors and supervisors were given one day training on data collection procedures. Prior to data collection, the check list was tested to check the consistency of the checklist format and the ability of the data collector’s performance. Pre-test was conducted on 5% of the cases that had CS at Addis Alem primary hospital then the checklist was modified based on the pretest results. The final checklist was checked by data collectors & supervisors on daily basis for completeness, accuracy, validity and consistency of data. The principal investigator and supervisors made day to day onsite supervision during the whole period of data collection.

## Result

### Socio-demographic characteristics of the mothers

**Fig. 1:**
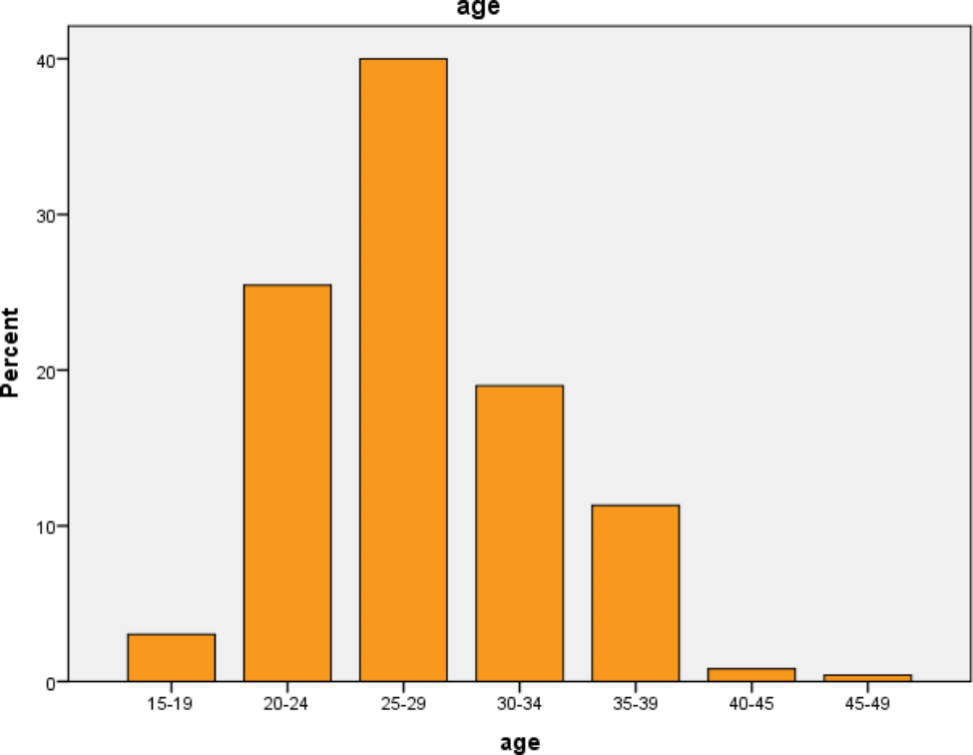
: Socio demographic characteristics of mothers who deliver via CS at Bahir Dar city public specialized hospitals, Bahir Dar, Ethiopia, 2021

The mean age for the mothers was 27.31 years with standard division of ± 4.986 years (Fig. 1). Two hundred nighty nine (60.4%) of the mothers were urban dwellers while 196 (39.6%) of them were from rural area.

### Maternal obstetric data and complications

51.3% of the women were multiparas (Para II - IV). Cesarean section was done at gestational age of 37 to 42 weeks or at term for 376 (76%) of the women. History of obstetric complication 289 (58.4%) of the mother had one or more obstetric complications during their pregnancy period (Table 1).

**Table 1:** Obstetrics data of the mothers who deliver via CS at Bahir Dar city public specialized hospitals, Bahir Dar, Ethiopia 2021

		Frequency	Percent
ANC	One or more visits No Visit	479 16	96.8 3.2
Parity	1 2–4 5–9 10 and above	209 254 29 3	42.2 51.3 5.9 0.6
GA	Unknown < 37 weeks 37–41 + 6 weeks 42 and above	60 28 376 31	12.1 5.7 76.0 6.3
Medical Comorbidity during pregnancy	No medical illness DM Obesity HIV/AIDS Others	466 10 10 6 6	94.1 2.02 2.02 1.2 1.2
History of CS scar	Yes No	158 337	31.9 68.1
Obstetrics Complication	No	206	41.6
	YES PROM APH PE/E Macrosomia Prolonged labor Malpresentation Multiple gestation Others	289 63 25 65 27 74 42 16 36	58.4 12 5.05 13.1 5.45 14.9 8.48 3.23 7.27

### Labor status of mothers

**Fig. 2:**
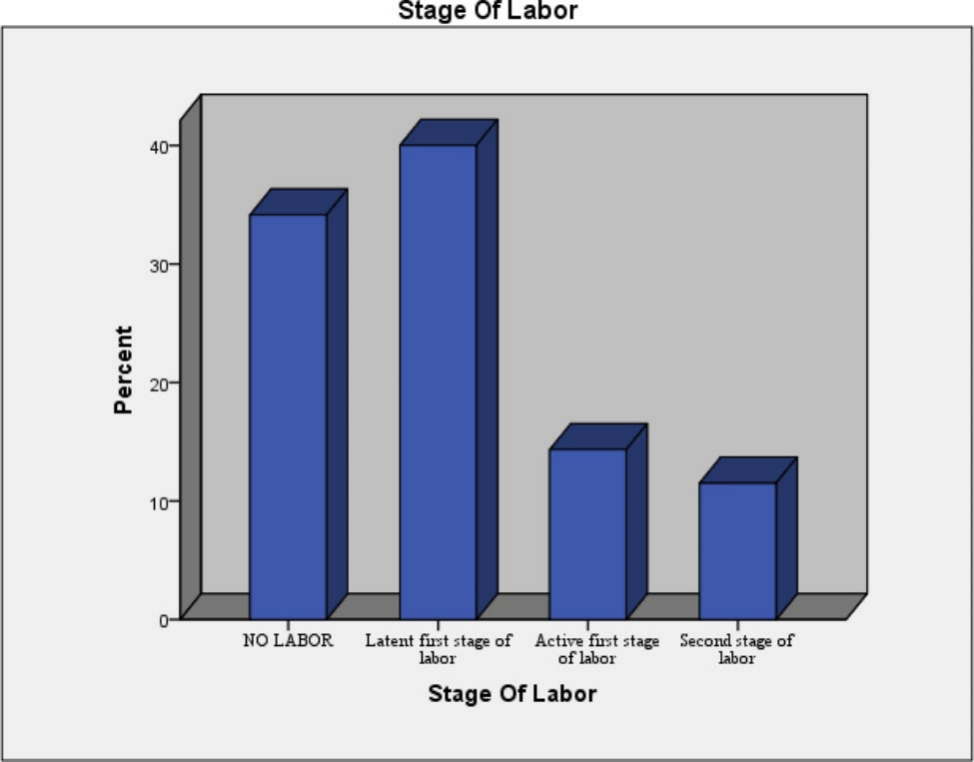
Labor status of mothers who deliver via Cesarean section at Bahir Dar city public specialized hospitals, Bahir Dar, Ethiopia, 2021

Three hundred eighty four (77.6%) of the women were operated in emergency basis and 111 (22.4%) of the cases were elective. Of emergency cases one hundred nighty eight (40%) of the women were operated at latent first stage of labor (Fig. 2).

### Intra-operative profile

Four hundred seventy three (95.6%) of the mothers were operated under spinal anesthesia and 487 (98.4%) were delivered by lower uterine segment incision. Time for accomplishing the operations shows 253 (51.1%) were completed within 30 to 60 min, 73 cases (14.7%) took more than 60 min, the maximum being 120 min (Table 2).

**Table 2:** Profile of cesarean section of the mothers who deliver via CS at Bahir Dar city public specialized hospitals, Bahir Dar, Ethiopia 2021

		Frequency	Percent
Type of incision	LUST Classical J shaped	487 7 1	98.4 1.4 0.2
Type of Anesthesia	SA GA	473 22	95.6 4.4
Duration of Surgery	< 30 30–60 > 60	169 253 73	34.1 51.1 14.7
Timing of surgery	Emergency Elective	384 111	77.6 22.4

### Intra-operative and post-operative surgical complications

Among mothers there were 53(10.7%) intra-operative and 197(39.8%) post-operative surgical complications. Among mothers the proportion of severe anemia 1.41% and moderate anemia 15.35% (Table 3).

**Table 3:** Intra - operative and post - operative complications of mothers who deliver via CS at Bahir Dar city public specialized hospitals, Bahir Dar, Ethiopia, 2021

Complication		Frequency	Percent
Intraoperative Complications	No	442	89.3
	Yes Incision extension Hemorrhage Adjacent organ injury Hysterectomy for intra-op bleeding Others	53 7 18 1 1 26	10.7 1.4 3.6 0.2 0.2 5.3
Postoperative complication	No	298	60.2
	Yes Fever Endomyometritis SSSI Anemia PPH RT infection Wound dehiscence DVT Re-laparotomy	197 10 5 23 182 12 3 1 1 2	39.8 2.0 1.0 4.6 36.8 2.4 0.6 0.2 0.2 0.4
Anemia Severity of Anemia	No	313	63.23
	Yes Mild Moderate Severe	182 99 76 7	36.77 20.00 15.35 1.41

#### Overall maternal complication

Two hundred eighteen of the mothers had at least one of the intra-operative or post-operative maternal complications. This makes the overall rate of complication among mothers who delivered by Caesarean section in TGSH, FHCSH and APH 44% (95% CI 39.6–48.5).

### Factors associated with maternal complications

In mutivariable logistic regression the factors found to be associated with maternal complication were rural residency, Previous CS scar, Having one or more obstetric complication, stage of labor, type of cesarean section, timing of cesarean section, gestational age and duration of the surgery (p value < 0.05).

Mothers who are living in rural area were 4 times more likely to develop complications of cesarean delivery (AOR = 4.247, 95%CI: 2.765, 6.522) as compared to urban areas.

Mothers who had having one or more obstetric complication were 2 times more likely to develop complications of cesarean delivery (AOR = 1.913, 95%CI: 1.214, 3.015) than the counterpart.

Mothers who operated in the second stage of labor were 4 times more likely to develop complications of cesarean delivery (AOR = 4.358, 95%CI: 1.841, 10.317) than as compared to not at labor stage.

Mothers who had history of previous cesarean section were 4 times more likely to develop complications of cesarean delivery (AOR = 3.540, 95%CI: 2.121, 5.910) than no history of CS.

Mothers who had emergency operation were 3 times more likely to develop complications of cesarean delivery (AOR = 2.967, 95%CI: 1.492, 5.901) than elective operations.

Mothers whose duration of CS more than one hour were 4 times more likely to develop complications of cesarean delivery (AOR = 3.476,95%CI: 1.521, 7.947) than duration of CS less than one hour (Table 4).

**Table 4:** Bivariate and multivariate logistic regression on maternal complications of women who deliver via CS at Bahir Dar city public specialized hospitals, Bahir Dar, Ethiopia, 2021

Variable	Maternal complication		COR 95% CI	AOR 95% CI	P value
	Yes	No			
**Residence**					
Urban	90	209	1	1	
Rural	128	68	4.371,(2.977,6.419)	4.247,(2.765, 6.522)	< 0.001
**obstetric complication**					
Yes	143	146	1.711(1.187,2.466)	1.913,(1.214, 3.015)	0.005
No	75	131	1	1	
**Stage of labor**					
No labor	61	108	1	1	
SSOL	43	14	0.184(0.093,0.363)	3.129,(1.330,7.361)	0.009
**Previous CS**					
Yes	82	76	1.595(1.090,2.333)	3.540,(2.121,5.910)	< 0.001
No	136	201	1	1	
**Duration of surgery**					
< 60	154	83	1	1	
≥ 60	62	50	3.564(1.731,7.335)	3.476,(1.521,7.947)	0.003
**Timing of surgery**					
Emergency	188	196	2.590(1.628,4.120)	2.967,(1.492,5.901)	0.002
Elective	30	81	1	1	

1 – Reference, COR-Crude Odds Ratio, CI- Confidence interval, AOR-Adjusted Odds Ratio

## Discussion

The overall maternal complications were 44.04% (CI 95% 39.6–48.5). This finding is higher compared to other studies conducted in Debre Birhan (16.5%) [16], Jimma (28%) [12], Finote Selam (28%) [13], Oromia (20.5%) [14], Tigray (19.3%) [15], Yirgalem (30.1%) [17] and Arbaminch (38.2%) [18]. It is also higher than the finding in Germany (10.5%) [19], But it was relatively lower than the finding in Hawassa which was found to be 56% [20]. The great difference might be due to anemia which was included as one of post-operative complication in this study but not in the previous studies. It can also be due to the difference in the nature of obstetric emergency in different areas and also most of the patients were referred cases with complicated deliveries which resulted in maternal complication.

Intra-operative surgical complications included hemorrhage 18 (3.6%), incision extension 7(1.4%), and adjacent internal organ injury 1 (0.2%). All the results were lower than the study findings in Arbaminch [18] and Tikur Anbessa [20] hospital, Ethiopia. The overall intraoperative complication was 10.7% which was lower than the previous two studies. This could be because the majority of the patients had ANC follow-up, for which most mothers were operated at the latent first stage of labor, and 68.1% of the surgeries in our study were primary CS with less medical comorbidity. 32 (6.46%) of the mothers had adhesion from previous surgery including minimal and dense adhesion which is comparable with study done in Ayder Specialized Comprehensive Hospital, Mekelle, Ethiopia(8.3%) [21]. Twenty three (4.6%) of women developed post-operative wound infection. The rate of wound infection was lower than the finding in Tigray Region (7%), Arbaminch (12%), Ayider (6.8%) and Jimma Hospital, Ethiopia (27.1%) [12, 15, 18, 21]. From the study done in Arba Minch, the justification for this could be the difference in sterility technique and choice of prophylaxis antibiotics among the hospitals [18].

Other complications include Anemia in 182 (36.8%) of the mothers, febrile morbidity among 10 (2.02%), PPH 12(2.4%), DVT among 1 (0.2%), Endomyometritis in 5 (1.01%), transfusion 0.8% of the mothers which defers from the result in Tigray region with PPH 1.68%, endometritis 13 (3.6%), transfusion 10 (2.8%), hysterectomy 6 (1.7%) [15]. Re-laparotomy was performed for 0.4% of the mothers which was 3% in both Ayider and Arba Minch, while it was found to be 6% in another study in Tigray [12, 15, 21].

The result of this research has showed that mothers living in rural settings are 4 times more likely to have maternal complications than those who live in urban areas. Cesarean section facilities are either not available or too far away for these women which render them to visit health facility after prolonged labor or after they experience at least one of obstetric complications which exposes the mother for complications. This finding was supported by previous studies done in Yirgalem and Oromia region, Ethiopia, which showed that rural women were 3 and 1.3 times more vulnerable for complication respectively [14, 17].

Mothers who had one or more obstetric complications were 2 times more likely to develop complications of cesarean delivery (AOR = 1.913, 95%CI: 1.214, 3.015) than the counterpart. This was consistent with the study in Arba Minch, Ethiopia [18]. The possible reason might be associated with the increased risk of emergency, surgery hemorrhage and intraoperative difficulties in women with complicated pregnancy.

Mothers who had emergency operation were 3 times more likely to develop complications of cesarean delivery than elective operations. This was supported by the research done in Nepal on maternal and fetal outcome in emergency versus elective Caesarean Section which concluded that maternal complications like post-operative wound infection, PPH, UTI, need for blood transfusion, post-operative fever in emergency CS were significantly higher than that in elective CS group [22]. This can be explained by poor pre-operative patient and operation theater optimization during emergency surgeries.

Mothers who had history of previous cesarean section were 4 times more likely to develop complications of cesarean delivery than no history of CS. It was supported by a systematic review and meta-analysis study on correlation between previous caesarean section and adverse maternal outcomes which found that previous CS was found to be associated with adverse maternal outcomes in subsequent pregnancy and childbirth [23]. This raise in complication risk can be associated with the intraoperative difficulties from adhesion from previous surgeries, excessive bleeding and increased risk of hysterectomy from increased incidence of uterine rupture in those with previous scar.

Mothers who operated in the second stage of labor were 4 times more likely to develop complications of cesarean delivery than as compared to not at labor stage. This was supported by the research done in Arba Minch, Ethiopia [18]. The reason may be from increased risk of hemorrhage, incision extension and major vessel involvement during second stage surgeries which resulted from deep impaction of fetal head and difficulty of extraction.

Mothers whose duration of CS was more than one hour were 4 times more likely to develop complications of cesarean delivery than duration of CS less than one hour. This was supported by the research done in Israel. There was a strong association between prolonged CD and post-operative adverse maternal outcomes including post-operative blood transfusion (4.4% vs. 1.5%), prolonged hospitalization (8.4% vs. 4.0%), infection necessitating antibiotic treatment (2% vs. 1%) and readmission (1.8% vs. 0.8%). This can be associated with the increased bleeding, manipulation and increased risk of infection associated with prolonged surgical time.

## Conclusion

The magnitude of maternal complication of cesarean section in this study is higher compared to studies conducted in Ethiopia (Debre Birhan, Jimma, Finote Selam, Oromia, Tigray, Yirgalem, and Arbaminch). Living in rural setting, having obstetric complications, previous cesarean scar, emergency surgeries, operation done in second stage of labor and prolonged duration of surgery are important predictors of maternal complication. Therefore, we recommend timely and adequate progress of labor evaluation, timely decision for cesarean delivery and vigilant care in post-operative period shall be conducted.

## Data Availability

The datasets used and/or analyzed during the current study are available from the corresponding author on reasonable request.

## List of abbreviations

AFSOL: Active First Stage Of Labor
APH: Addisalem Primary Hospital
CD/CS: Cesarean Delivery/ Section
CPD: Cephalo pelvic Disproportion
DM: Diabetes Mellitus
DVT: Deep Venous Thrombosis FHRH–Felege Hiwot Referral Hospital
GA: Gestational Age
HTN: Hypertension
LFSOL: Latent First Stage Of Labor
NRFHRP: Non-Reassuring Fetal Heart Rate Pattern
PPH: Post-Partal Hemorrhage
PROM: Premature Rupture Of Membrane
SNNPR: Southern Nations Nationalities And People
SSOL: Second Stage Of Labor
TGSH: Tibebe Ghion Specialized Hospital
